# Evaluation of the Edinburgh Post Natal Depression Scale using Rasch analysis

**DOI:** 10.1186/1471-244X-6-28

**Published:** 2006-06-12

**Authors:** Julie F Pallant, Renée L Miller, Alan Tennant

**Affiliations:** 1 Faculty of Life and Social Sciences, Swinburne University of Technology, P.O. Box 218, Hawthorn, Victoria 3122, Australia; 2 Academic Unit of Musculoskeletal & Rehabilitation Medicine, The University of Leeds, 36 Clarendon Road, Leeds. LS2 9NZ, UK

## Abstract

**Background:**

The Edinburgh Postnatal Depression Scale (EPDS) is a 10 item self-rating post-natal depression scale which has seen widespread use in epidemiological and clinical studies. Concern has been raised over the validity of the EPDS as a single summed scale, with suggestions that it measures two separate aspects, one of depressive feelings, the other of anxiety.

**Methods:**

As part of a larger cross-sectional study conducted in Melbourne, Australia, a community sample (324 women, ranging in age from 18 to 44 years: mean = 32 yrs, SD = 4.6), was obtained by inviting primiparous women to participate voluntarily in this study. Data from the EPDS were fitted to the Rasch measurement model and tested for appropriate category ordering, for item bias through Differential Item Functioning (DIF) analysis, and for unidimensionality through tests of the assumption of local independence.

**Results:**

Rasch analysis of the data from the ten item scale initially demonstrated a lack of fit to the model with a significant Item-Trait Interaction total chi-square (chi Square = 82.8, df = 40; p < .001). Removal of two items (items 7 and 8) resulted in a non-significant Item-Trait Interaction total chi-square with a residual mean value for items of -0.467 with a standard deviation of 0.850, showing fit to the model. No DIF existed in the final 8-item scale (EPDS-8) and all items showed fit to model expectations. Principal Components Analysis of the residuals supported the local independence assumption, and unidimensionality of the revised EPDS-8 scale. Revised cut points were identified for EPDS-8 to maintain the case identification of the original scale.

**Conclusion:**

The results of this study suggest that EPDS, in its original 10 item form, is not a viable scale for the unidimensional measurement of depression. Rasch analysis suggests that a revised eight item version (EPDS-8) would provide a more psychometrically robust scale. The revised cut points of 7/8 and 9/10 for the EPDS-8 show high levels of agreement with the original case identification for the EPDS-10.

## Background

The Edinburgh Postnatal Depression Scale (EPDS) [1] is a 10 item self-rating post-natal depression scale which was developed almost twenty years ago. Its 10 polytomous items are summated to an overall score ranging from 0–30, with cut points to identify the likely presence of depression. It has seen widespread use in epidemiological and clinical studies [2-4]. Although originally intended as a measure of depression, a number of authors have speculated that it may be measuring something more general. Green [5] suggests that, given its high correlation with a variety of other measures, the EPDS may be measuring what she refers to as postnatal 'dysphoria' (p.153).

Concern has also been raised over the validity of the EPDS as a single unidimensional summed scale, with suggestions that it measures two separate aspects, one of depressive feelings, the other of anxiety [6-9]. Brouwers et al. [7], for example, identified two subscales using exploratory factor analysis (EFA), representing anxiety (items 3,4,5) and depression (items 1,2,8), with the two subscales showing only a moderate correlation of .37. This result was confirmed by Jomeen and Martin [10] who also found a separation of anxiety and depression items in both EFA and confirmatory factor analysis (CFA). CFA assessment of the unidimensional model as proposed by Cox recorded the worst model fit statistics, when compared with the alternative multidimensional models proposed by Brouwers et al. [7] and Ross et al. [8]. In each of these studies item 10 (*The thought of harming myself has occurred to me*) loaded on a third and separate factor and was not included in subsequent analyses. A three factor solution was identified in a study by Chabrol and Teissedre[11], distinguishing anxiety, depressive mood and anhedonia.

The majority of scales in the health and social sciences have been developed using traditional psychometric approaches involving the assessment of validity and reliability [12]. Construct validity has often been supported through factor analytic techniques which confirm the presence of one or more valid unidimensional scales. Unfortunately, rating scales give ordinal data which fail to meet the assumptions of parametric factor analysis, and it is known that the misuse of the technique can lead to incorrect interpretations [13]. However these traditional techniques are now being complemented and, in some cases replaced, by Item Response Theory approaches and particularly by the application of the Rasch measurement model [14-19].

This paper examines the potential contribution of Rasch analysis in understanding measurement issues associated with the EPDS. In particular it addresses the question: does the scale provide a psychometrically valid single unidimensional measure of post natal depression, or are there two subscales within its ten items? [1,9]. In addition it explores the appropriateness of the response format used, and assesses the potential bias of items by age and educational level of the mother, and age of the child.

## Methods

### Participants

A total of 324 women, ranging in age from 18 to 44 years (mean = 32 yrs, SD = 4.6), participated in this study. The age of women's babies at the time of completing the questionnaire ranged from 6 weeks to 6 months, with a mean age of 13 weeks (SD = 5.0). The majority of women (94%) were married (n = 248) or in a defacto relationship (n = 59), with 9 women (2.8%) in a non-cohabiting relationship, 5 women (1.5%) were single, 2 women (0.6%) were divorced, and 1 woman (0.3%) was widowed. 103 women (31.9%), reported having had no tertiary education, 107 women (33.1%) had completed undergraduate university degrees, and 113 women (34.8%) had completed postgraduate university degrees.

### Procedure

As part of a larger cross-sectional study[20] conducted in Melbourne, Australia, a community sample was obtained by inviting primiparous women (recruited primarily through mothers' groups) to participate voluntarily in this study. The study was approved by the Swinburne University of Technology Ethics Committee. Women were asked to complete a questionnaire and return it via post, with no identifying information included. In order to reduce the potential confounds of additional children, criteria for inclusion limited participants to first time mothers with no step or foster children. Participants were required to be between 6 weeks and 6 months postnatal.

### Rasch analysis

Data are fitted to the Rasch model using the RUMM2020 software [21]. According to Linacre [22] if a scale is well targeted (i.e. 40–60% endorsement rates on dichotomous test items) then a sample size of 108 will give 99% confidence of the person estimate being within ± 0.5 logits. If the scale is not well targeted (i.e. < 15% or > 85% endorsement rate), then the sample size required for accurate estimation increases to 243. Consequently the sample size of 324 women in the current study is large enough to give good precision, regardless of the targeting of the sample (the relationship between the distribution of persons and the distribution of items on the same metric scale).

The Rasch methodology adopted in this study is described in detail elsewhere [23]. Briefly, the Rasch model [24] is seen as a template which puts into operation the formal axioms which underpin additive conjoint measurement [25]. Dichotomous [24] and polytomous [26] versions of the model are available and a further variant of the latter, which is used in this paper, is known as the partial credit model [27]:

ln⁡(Pnij1−Pnij−1)=θn−bij
 MathType@MTEF@5@5@+=feaafiart1ev1aaatCvAUfKttLearuWrP9MDH5MBPbIqV92AaeXatLxBI9gBaebbnrfifHhDYfgasaacH8akY=wiFfYdH8Gipec8Eeeu0xXdbba9frFj0=OqFfea0dXdd9vqai=hGuQ8kuc9pgc9s8qqaq=dirpe0xb9q8qiLsFr0=vr0=vr0dc8meaabaqaciaacaGaaeqabaqabeGadaaakeaacyGGSbaBcqGGUbGBdaqadaqaamaalaaabaGaemiuaa1aaSbaaSqaaiabd6gaUjabdMgaPjabdQgaQbqabaaakeaacqaIXaqmcqGHsislcqWGqbaudaWgaaWcbaGaemOBa4MaemyAaKMaemOAaOMaeyOeI0IaeGymaedabeaaaaaakiaawIcacaGLPaaacqGH9aqpiiGacqWF4oqCdaWgaaWcbaGaemOBa4gabeaakiabgkHiTiabdkgaInaaBaaaleaacqWGPbqAcqWGQbGAaeqaaaaa@4939@

where P_*nij *_is the probability that person *n *will answer affirm category *j *of item *i *[or be able to do the level of a task specified by that category within the item], *θ *is person ability, and *b *is the item difficulty parameter. From this, the expected pattern of responses to an item set is determined given the estimated *θ *and b. The expected pattern is a probabilistic form of Guttman scaling [28], and a variety of fit statistics determine if this is the case [29]. Three overall fit statistics are considered. Two are item-person interaction statistics transformed to approximate a z-score, representing a standardized normal distribution where perfect fit to the model would have a mean of approximately zero and a standard deviation of 1. A third is an item-trait interaction statistic reported as a Chi-Square, reflecting the property of invariance across the trait. A significant Chi-Square indicates that the hierarchical ordering of the items varies across the trait, so compromising the required property of invariance.

In addition to these overall summary fit statistics, individual person- and item fit statistics are presented, both as residuals (a summation of individual person and item deviations) and as a chi-square statistic. In the former case residuals between ± 2.5 are deemed to indicate adequate fit to the model. In the latter case a chi-square fit statistic is available for each item, and the overall chi-square for items is summed to give the item trait-interaction statistic. An estimate of the internal consistency reliability of the scale is also available, based on the Person Separation Index (PSI) where the estimates on the logit scale for each person are used to calculate reliability.

Sources of deviation from model expectation are examined to see if the scale construct can be improved. For a good fitting model we would expect that, for each of the items, respondents with high levels of the attribute being measured would endorse high scoring responses, while individuals with low levels of the attribute would consistently endorse low scoring responses. In Rasch analysis terms this would be indicated by an ordered set of response thresholds for each of the items. The term *threshold *refers to the point between two adjacent response categories where either response is equally probable. For a given item the number of thresholds is always one less than the number of response options. Disordered thresholds occur when respondents have difficulty consistently discriminating between response options. This can occur when there are too many response options, or when the labelling of options is confusing. Usually, although not always, collapsing of categories where disordered thresholds occur improves overall fit to the model.

Another issue that can affect model fit is differential item functioning (DIF). This occurs when different groups within the sample (e.g. males and females), despite equal levels of the underlying characteristic being measured, respond in a different manner to an individual item. For example men and women with equal levels of depression may respond systematically differently to an item in a depression inventory [30]. Two types of DIF may be identified. Uniform DIF is where the group shows a consistent systematic difference in their responses to an item, across the whole range of the attribute being measured, and non-Uniform DIF is where differences vary across levels of the attribute. Analysis of variance is conducted for each item comparing scores across each level of the 'person factor' (eg. gender) and across different levels of trait (referred to as class intervals). Uniform DIF is indicated by a significant main effect for the person factor (gender), while the presence of non-uniform DIF is indicated by a significant interaction effect (person factor X class interval).

Finally, when issues of threshold disordering, DIF and fit have been resolved a Principal Components Analysis (PCA) of the residuals detects any signs of multidimensionality. After the 'Rasch' factor has been extracted there should be no associations left in the data. There are several ways to detect this, including the proportion of variance attributable to the first residual factor compared with that attributable to the first (Rasch) factor, and whether or not estimates derived from subsets of items are invariant (specific objectivity). This latter is formally tested by allowing the factor loadings on the first residual to determine 'subsets' of items and then testing, by a paired *t*-test, to see if the person estimate (the logit of person 'ability' or, in this case 'depression') derived from these subsets significantly differs between subsets [31]. If the person estimate is found to differ between the subsets of items this would indicate the presence of multidimensionality. An effect size for the difference can also be calculated to determine the substantive nature of such a difference.

Where the data fit the model, and the assumptions of local independence are met, a unidimensional linear scale is derived from the ordinal raw score, thus opening up the opportunity to validly apply parametric approaches [32,33]. Thus, fitting data to the Rasch model offers a useful approach to addressing key methodological aspects of scale development, including dimensionality, category ordering and item bias.

## Results

Rasch analysis of the data from the ten item scale using RUMM2020 showed a lack of fit to the Rasch model with a significant Item-Trait Interaction total chi-square (chi-square = 82.8, df = 40; p < .001). The mean residual for items was -0.50 with a standard deviation (SD) of 1.575, whereas the latter would be expected to be much closer to 1, given adequate fit to the model. The mean residual for persons was -0.287 with a SD of 0.855, indicating no serious misfit among the respondents in the sample.

**Figure 1:**
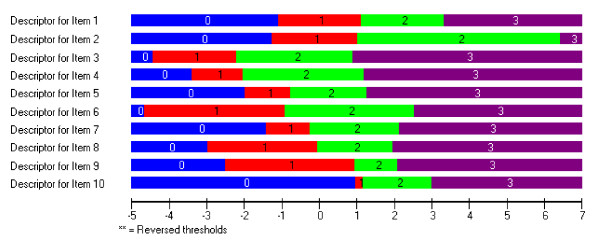
Threshold Map for 10-item EPDS.

Initially, the pattern of thresholds was examined to see if disordering may be affecting fit. In the current example all thresholds were ordered (Figure 1). The threshold distances vary across items (see varying lengths of category one across items), supporting the use of the partial credit model for the analysis of this scale. A log likelihood ratio test statistic confirmed that this was the case (p < 0.001).

**Figure 2:**
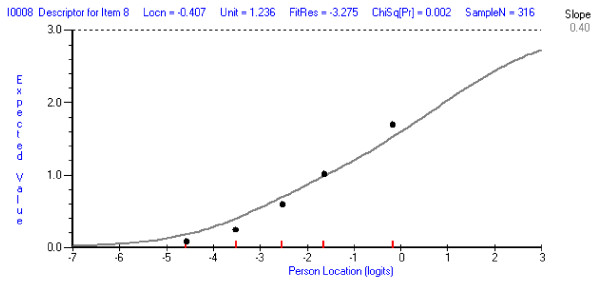
Fit of item 8 *I have felt sad or miserable.*

Two items initially showed misfit to model expectations, Item 8 (*I have felt sad or miserable*) and Item 5 (*I have felt scared or panicky for no very good reason*) (see Table 1). Item 8 showed a Fit Residual value of -3.275 and a chi-square probability value of 0.002, less than the Bonferroni adjusted alpha value of .005, indicating significant deviation from the model expectation. The negative Fit Residual value obtained suggests a high level of discrimination, shown by the ICC for the item where observed responses are steeper than the expected curve (Figure 2). Thus responses from the lowest group (low levels of depression) are below what is expected by the model and those for the highest group (high levels of depression), are above model expectation. This high negative residual is usually associated with dependency, and a high item-total correlation, signifying redundancy of the item.

**Table 1:** Item fit statistics

Item	Location	SE	FitResid	DF	ChiSq	DF	Prob
1. I have been able to laugh and see the funny side of things	1.067	0.125	-0.993	281.46	3.26	4	0.515
2. I have looked forward with enjoyment to things	2.429	0.125	-0.315	280.57	2.62	4	0.623
3. I have blamed myself unnecessarily when things went wrong	-1.958	0.094	1.958	280.57	13.52	4	0.009
4. I have been anxious or worried for no good reason	-1.456	0.089	0.843	281.46	4.62	4	0.329
**5. I have felt scared or panicky for no very good reason**	**-0.541**	**0.094**	**0.794**	**281.46**	**15.84**	**4**	**0.003**
6. Things have been getting on top of me	-1.069	0.109	-1.162	279.67	8.10	4	0.088
7. I have been so unhappy that I have had difficulty sleeping	0.118	0.104	-0.935	281.46	5.34	4	0.255
**8. I have felt sad or miserable**	**-0.407**	**0.103**	**-3.275**	**281.46**	**17.55**	**4**	**0.002**
9. I have been so unhappy that I have been crying	0.133	0.112	-2.364	281.46	7.86	4	0.097
10. The thought of harming myself has occurred to me	1.684	0.174	0.349	281.46	4.15	4	0.386

Misfitting items are shown in bold.

Removal of Item 8 led to an improvement in fit to the model with a non-significant (Bonferroni adjusted) Item-Trait Interaction total chi-square (chi-square = 60.2, df = 36, p = 0.007). The Residual mean value for items became -0.47 with a standard deviation (SD) of 0.909, showing much better fit to the model. Individual person fit statistics showed that no respondents had residuals outside the acceptable range for the 9-item solution. Following the removal of item 8, individual item fit statistics were again reviewed, and item 5, which initially showed misfit to the model, now showed a response pattern consistent with model expectation, and was therefore retained.

**Figure 3:**
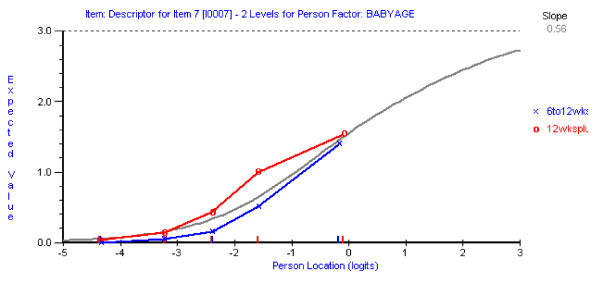
Differential item functioning for age of baby for Item 7.

In the 9-item solution the possibility of item bias was explored for the age of the mother, educational level of the mother, and the age of the child, using a Bonferroni adjusted p value of 0.003 (0.05/18). Just one of the items Item 7 (*I have been so unhappy that I have had difficulty sleeping*) recorded a probability value exceeding the adjusted alpha value, showing some degree of uniform DIF for age of child (see Figure 3). Inspection of the DIF graph suggests that, at equal levels of depression, mothers with very young babies (6 to 12 weeks) are less likely to endorse this item. As DIF is a breach of unidimensionality, this item was also deleted. This gave a non-significant (Bonferroni adjusted) Item-Trait Interaction total chi-square (chi-square = 53.8, df = 32, p = 0.009). The Residual mean value for items was -0.467 with a standard deviation (SD) of 0.850, showing fit to the model. No DIF now existed in this 8-item scale (EPDS-8) and all items showed fit to model expectations.

**Figure 4:**
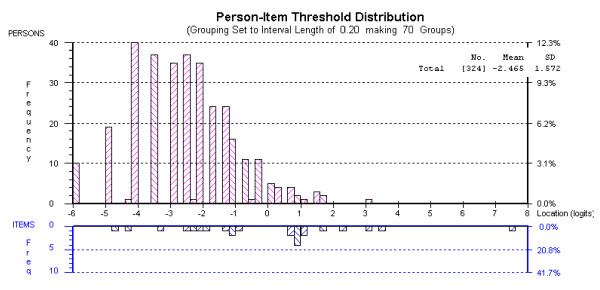
Targeting map for 8-item EPDS.

Figure 4 shows the distributions of persons and item thresholds of the revised scale, with persons on the upper part of the graph, and the item thresholds on the lower part. The average mean person location value of -2.465 suggests that the respondents were well below the average of the scale. However, for a screening instrument this is not necessarily of great concern, as the cut point for a clinical case is the key issue. The PSI Statistic was 0.804, which indicates that the scale has adequate person separation reliability.

A principal component analysis of the residuals revealed a first residual factor accounting for 1.8% of the total variance in the data, or 22% of the variance in the residuals. Two sets of items were found to load positively and negatively on the first residual component. A paired t-test indicated that neither of these two sets gave a person estimate significantly different to the other (p = 0.14) and the effect size of the difference was 0.08. Consequently the assumption of local independence is upheld, and the EPDS-8 can be considered to be a unidimensional scale.

To determine cut points on this revised 8-item scale individuals were first classified according to the original 10-item EPDS cut points [1]. This allowed each person to be identified as not depressed (range 0–9); minor depression (range 10–12) or more major depression (range 13 or more). For minor depression a cut point of 8 or more on the EPDS-8 maximised the kappa (0.9), identifying 95% of those classified as such by the original 10-item scale. This cut point also identified 96.7% of those identified as not depressed by the original scale. For major depression a cut point of 9 or more on the EPDS-8 identified all those so classified by the original and 91.9% of those without major depression, but the kappa was lower (0.71) than a cut point of 10+ (0.86) which identified 97.2% of those classified as having major depression on the original, and 96.8% of those without major depression.

**Figure 5:**
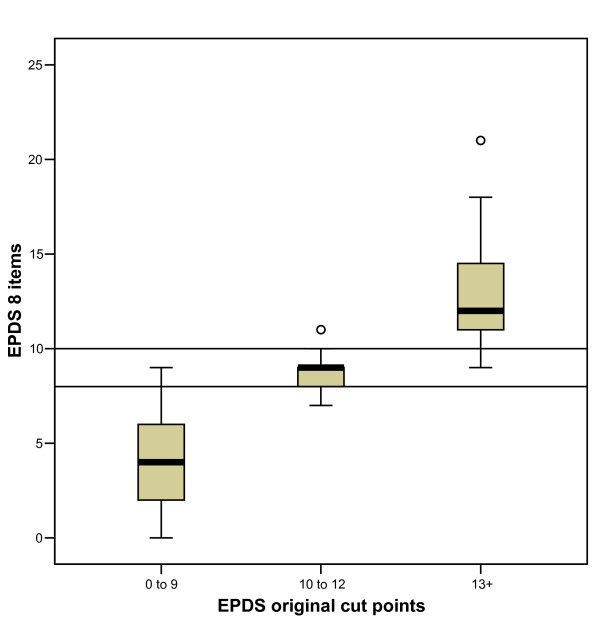
Boxplot showing 8-item EPDS scores for women classified into groups using the original EPDS cutpoints (scores 0 to 9, 10 to 12, 13+).

Figure 5 shows the distribution of scores on the EPDS-8 for each group classified using the original EPDS. The cut point of 8 or more for minor depression, and 10 or more for major depression (shown as the horizontal lines on the graph) clearly separates cases with no evidence of depression, as defined by the original scale, from those with minor and major depression (Kruskal-Wallis: chi-square = 179.1; df = 2; p < .001).

The results of the above analysis suggest that an eight item version of the scale would be more psychometrically robust, in that it would be free of item bias caused by the influence of baby age on Item 7, and also removes Item 8 which showed misfit to the model. It also has high levels of agreement with the original case identification. The scale has an approximate linear range only for the raw score range of 4 to 20 (from a range of 0–24 on the EPDS-8).

## Discussion

Despite its widespread use, the viability of the original 10-item EPDS has been found to fall short of the rigorous standards of measurement defined by the Rasch model. The use of Rasch analysis in this study has enabled a detailed examination of the structure and operation of the scale. The ordering of response categories (threshold ordering) has not been examined previously, and the evidence from this current study supports the response format used, but not the validity of the full original 10-item scale. It was necessary to remove two items from the scale, in order to achieve model fit. Item 7 (*I have been so unhappy that I have had difficulty sleeping*) was removed because it showed differential item functioning for the age of the baby. Although there are techniques to accommodate uniform DIF by allowing the item difficulty to vary by group [34], we thought this inappropriate as this option is not practical in an everyday screening environment. Consequently we chose to delete the biased item (Item 7). Inspection of the item wording revealed that this item is potentially confusing as it mixes the concepts of unhappiness with sleep, which may be confounded by the mother's expectations, and/or the child's lack of sleep. This could be one reason why the item works differently according to the age of the child. Removal of this item from the scale improved the overall model fit, supporting this decision.

Item 8 (*I have felt sad or miserable*) was also removed from the scale due to misfit to the model. At first sight this may seem a strange omission, in that the item appears to have face validity. The high negative residual misfit indicates that it adds nothing to the information gained by other items, and its removal significantly improved the fit of those remaining items. In some respects it is more like a summary item with a high item-total correlation (0.8). Further research is needed to assess the replicability of this finding in other samples. The results of this study support the retention of Item 10 *The thought of harming myself has occurred to me*, despite previous factor analytic studies which led to its exclusion. Its low endorsement rate and item-total correlation may have contributed to this, as it is known that factor analysis can misidentify factors by frequency (92% scored a value of zero on this item).

The results of this study do not support the structure of the original 10-item scale as proposed by the scale developers [1]. However there is also no evidence supporting the alternative structure identified by Brouwers et al. [7], Ross et al. [8] and Pop et al. [9] separating the anxiety (items 3,4,5) and depression items (items 1,2,8). Although two sets of items were identified in PCA of the residuals, the person estimates (Rasch logit-based estimate) derived from the subsets were not significantly different from one another, thereby supporting its unidimensionality.

Finally, the fit of the EPDS to the Rasch model has shown that the scale in its raw form is ordinal. This is not necessarily a problem when the scale is used with the cut points to identify those with depression, as an ordinal scale does just as well under these circumstances. However, depending on distribution of patients, there would be a problem if change scores needed to be calculated [32] and this would consequently need a Rasch transformed score.

The focus of this study has been the use of Rasch analysis to assess the measurement properties of the EPDS in terms of its structure, item fit and freedom from bias. This does not however provide a test of the clinical validity of the scale. Further studies are required to formally assess the revised format of the scale (EPDS-8) in clinical settings and to explore the appropriateness of the recommended cut-points using alternative assessment tools, such as standardized diagnostic interviews. The screening capacity of the shortened version of the EPDS identified in this study will need to be clinically assessed against the original 10-item EPDS.

## Conclusion

In summary, it would appear that the total EPDS, in its original 10-item form, is not a valid scale for the measurement of depression. The results of this study suggest that a revised eight item version, the EPDS-8, would provide a more psychometrically robust scale. The revised cut points of 7/8 and 9/10 for the EPDS-8 show high levels of agreement with the original case identification for the EPDS-10.

## Competing interests

The author(s) declare that they have no competing interests.

## Authors' contributions

JFP supervised the design of the study and the statistical analyses undertaken. RLM collected the data and helped to design the study. AT assisted with the analysis of the data and interpretation of the results. All authors contributed to the preparation of the article. All authors read and approved the final manuscript.

## Pre-publication history

The pre-publication history for this paper can be accessed here:


